# All my life to live: travel health benefits and risks for cancer survivors

**DOI:** 10.1093/jtm/taac069

**Published:** 2022-06-01

**Authors:** Jessica Hui Cheah Lim, Cian Keenan, Gerard Thomas Flaherty

**Affiliations:** Edinburgh Cancer Research Centre, Institute of Genetics and Cancer, University of Edinburgh, Edinburgh, UK; School of Medicine, National University of Ireland Galway, Galway, Ireland; School of Medicine, National University of Ireland Galway, Galway, Ireland; School of Medicine, International Medical University, Kuala Lumpur, Malaysia

**Keywords:** Cancer, chemotherapy, immunosuppression, stomas, travel insurance, travel health

## Introduction

Cancer is responsible globally for more than one in five of all deaths from non-communicable diseases. Cancer of the lung and stomach are among the top 10 causes of death in upper-middle-income countries, from where the bulk of international travellers originate.^1^ Travel itself may be regarded as a risk factor for some cancers, with a higher incidence of skin cancer occurring in fair-skinned travellers who sustain severe sunburn, and of breast cancer in female flight attendants.^2^ The incidence of cancers related to western lifestyles is generally lower, and those resulting from infectious diseases higher, in migrants than in host populations.^3^

Impressive advances in cancer care have greatly improved clinical outcomes with a strong focus on cancer survivorship and the enhancement of patients’ quality of life in partnership with multidisciplinary healthcare teams. As society emerges from the most devastating effects of the COVID-19 pandemic, which exacted a heavy toll on immunosuppressed cancer patients, we anticipate that levels of confidence towards international travel will gradually increase in the cancer survivor community. A recent study of websites aimed at patients living with a variety of chronic illnesses, including cancer, exposed significant deficiencies in the quality of travel health information they provide.^4^ Although there has been a traditional emphasis on infectious disease risk mitigation in immunocompromised travellers with cancer, this perspective will address the full spectrum of travel health risks in this population, but also anticipate the health benefits of travel in this group.

## Travel health benefits

The therapeutic effect of travel is an undeveloped concept in travel medicine journals and the few reports on the health benefits of international leisure travel have emerged from tourism studies research. Two decades have passed since a qualitative study was published on the perceived role of holiday-taking in the post-cancer rehabilitation and recovery of cancer patients. Patients, their families and carers identified multiple positive effects of leisure travel, including enhanced mood, a feeling of wellbeing from sunnier climates, improved mobility, increased energy levels, diminished social isolation, recovery of lost self-esteem, a more positive self-image, greater independence and an opportunity to escape from a burdensome sick role.^5^ We believe that patients with active cancer and cancer survivors should not be deprived of the health benefits of travel. Notwithstanding the modifiable travel health risks, which are addressed in a pre-travel consultation, healthcare professionals should encourage and facilitate their patients’ efforts to travel during stable phases of the cancer journey.

## Travel patterns of individuals with cancer

We have an incomplete understanding of the international travel patterns, travel behaviour and clinical outcomes of patients travelling with cancer. The travel health challenges faced by people with cancer usually focus on whether it is safe to administer live-attenuated vaccines such as yellow fever vaccine and infectious disease risks, including travellers’ diarrhoea and malaria, arising from the immunocompromising effects of malignancy and its treatment, including chemotherapy, biologic agents, radiotherapy and surgical splenectomy. A retrospective cohort study of 149 patients with diagnosed cancer presenting for pre-travel health advice found that 47% were immunocompromised at the time of travel with similar exposures and travel itineraries to patients whose cancer was cured or in remission. Most reported travel-related illnesses in that cohort were classified as minor.^6^ Only 56% of stem cell transplant (SCT) recipients in a cross-sectional study had attended for pre-travel health advice. The 7% of SCT patients who reported illness during travel were more likely to have visited high-risk areas, had a longer trip duration and visited friends and relatives.^7^ We could not identify any qualitative studies in the medical literature of cancer patients’ attitudes and experiences in relation to international travel. Such traveller-generated insights would be invaluable in tailoring advice to this group of travellers.

## Non-infectious travel health risks

The non-infectious risks of travel faced by cancer patients have received less attention in the literature and there is little evidence-based information to guide practitioners’ advice, which is still primarily based on expert opinion. Based on the authors’ (JHWL, GTF) combined clinical experience in medical oncology, patients often seek guidance on these issues when discussing international travel plans. Healthy behaviour during times of peak ultraviolet (UV) radiation exposure and skin protection with high sun protection factor cream are essential for cancer patients who have received chemotherapy. Depending on the phase of their cancer treatment, fatigue can be a significant barrier to normal daily activities and travel itineraries and modes of transportation may need to be adapted to the patient’s energy levels. The disfiguring cosmetic effects of cancer treatment, including surgical scars, hair loss, skin grafts and stomas can be a source of anxiety for travellers, especially if they are at an early stage of their cancer experience. The care of central venous catheters, percutaneous endoscopic gastrostomy (PEG) tubes and portacath lines in patients undergoing active cancer treatment will require specific advice from the patient’s oncologist or specialist cancer nurse. Travellers to high altitude who have received radiotherapy for head and neck cancer should be warned about their increased risk of acute mountain sickness arising from damage to the carotid bodies.

Depression is common among active cancer patients and cancer survivors and travelling with at least one supportive companion should be encouraged. Sleep may be disturbed and cancer patients may request sedative-hypnotic agents from their travel medicine provider. Melatonin may be an effective treatment for jet lag; it is a safe option in cancer patients and may even help to alleviate some chemotherapy side effects.^8^ We recommend that all travellers with a history of cancer join a peer support group. Many cancer patient organizations will provide emotional and practical support to travellers overseas via telehealth consultations and online communications. Selected web-based resources for cancer patients and their travel health advisers are provided in Table 1.

**Table 1:** Information resources for travellers with cancer and their health advisers

**Cancer patient organization**	**Online resource**
American Cancer Society Bowel Cancer UK BreastCancer.org Centers for Disease Control and Prevention Canadian Cancer Society Cancer Research UK Cancer.Net European Association for Cancer Research Hospiceinformation.info International Association for the Study of Lung Cancer International Gynaecologic Cancer Society Kidney Cancer Association Macmillan Cancer Support National Cancer Institute National Coalition for Cancer Survivorship No Stomach for Cancer Prostate Cancer UK Skin Cancer Foundation Testicular Cancer Society World Child Cancer	https://www.cancer.org/ https://www.bowelcanceruk.org.uk/ https://www.breastcancer.org/ https://wwwnc.cdc.gov/travel/ https://cancer.ca/en/ https://www.cancerresearchuk.org/ https://www.cancer.net/ https://www.eacr.org/ http://www.hospiceinformation.info/ https://www.iaslc.org/ https://igcs.org/ https://www.kidneycancer.org/ https://www.macmillan.org.uk/ https://www.cancer.gov/ https://canceradvocacy.org/ https://nostomachforcancer.org/ https://prostatecanceruk.org/ https://www.skincancer.org/ https://testicularcancersociety.org/ https://worldchildcancer.org/

## Fitness to travel

Communication between the traveller’s clinical oncology team and travel medicine clinician will facilitate informed decisions around patients’ fitness to travel. Cancer patients are likely to delay travel until cycles of chemotherapy and radiotherapy have been completed and bone marrow suppression has subsided, but occasionally travel may be prioritized for important family events or in situations where people have been displaced from their homeland by conflict or natural disasters. Under normal circumstances, travel insurance companies are unlikely to provide specialized cover for a traveller with cancer without a medical clearance certificate from the patient’s physician. Most routine travel insurance policies exclude pre-existing diseases, including cancer, so travellers may be required to purchase special cover for an extra premium and undergo medical screening.

Previous tracheostomy or laryngectomy should not be a barrier to air travel and patients may benefit from precautionary measures including keeping the breathing stoma moist, arranging for supplemental oxygen and carrying a portable nebuliser. Prevention of deep venous thrombosis will necessitate the use of graduated compression stockings and possibly use of low molecular weight heparin under specialist supervision. Bleeding risk in cancer patients with bone marrow suppression causing thrombocytopenia should be considered before prescribing antiplatelet or anticoagulant agents. Gas expansion at aircraft cruising altitude may increase the risk of wound dehiscence so minimum safe intervals following cancer surgery or laparoscopic procedures should be respected. This is a major consideration in cancer patients who have undergone neurosurgery because of the risk of expansion of gas in the skull. Changes in air pressure may result in increased gas in stoma bags, which may benefit from a flatus filter. Adhering to a healthy diet is essential to avoid increased intestinal output.

The incidence of lymphoedema exacerbation in cancer patients who have undergone surgical lymph node clearance is lower than previously suggested and this should not generally represent a barrier to air travel.^9^ Nonetheless, travellers with lymphoedema may require compression garments during a long journey and should be instructed to take prescribed antibiotics at the first appearance of cellulitis. Chronic jet lag is regarded as a risk factor for certain cancers and cancer patients taking regular medications should be informed about the appropriate timing of their medications when crossing time zones (east–west or west–east travel only). Transportation of prescribed controlled drugs such as narcotic analgesics for personal use should be informed by a knowledge of country-specific restrictions on quantities and accompanying documentation.^10^

## Accessing medical care overseas

Medical and surgical tourism is not uncommon among cancer patients, especially those living in smaller high-gross domestic product (GDP) countries in the Middle East such as Bahrain and the United Arab Emirates. In addition, cancer patients may travel overseas to enrol in a clinical trial or receive complementary and alternative medicine as part of a holistic programme of cancer survivor health care. A comprehensive review of the infectious risks of medical tourism has been recently published in this journal.^11^ The repatriation of large numbers of citizens to their home countries when COVID-19 was declared a global pandemic in early 2020 greatly complicated the continuity of care received by medical tourists with cancer. Cancer patients travelling abroad should be aware of any reciprocal care agreements between their origin and host countries (e.g. the European Health Insurance Card), but also recognize the limitations of such care, which may not cover non-emergency care and is not meant to be a substitute for comprehensive travel health insurance.

## End of life care in international travellers

For all travellers with cancer, their resuscitation wishes and advance directives should be made known to their travelling companions and clinicians who care for them abroad. Travellers should be aware that commercial airline flight attendants are unlikely to withhold basic life support in the event of a cardiorespiratory arrest occurring during flight in a passenger whose intentions not to be resuscitated have been communicated by their family members. This may cause considerable distress to the family of the traveller with cancer. For travellers with advanced cancer or receiving palliative care for a terminal diagnosis, education of accompanying persons about repatriation procedures in the event of a death occurring overseas is distressing but necessary.^12^ Suicide tourism may involve the non-physician-assisted taking of one’s life or physician-assisted euthanasia, the latter frequently witnessing the travel of patients with a terminal cancer diagnosis or debilitating neurodegenerative condition. A phenomenon exists whereby terminally ill people will prefer, for cultural reasons, to return to their country of birth to die. Travel medicine providers should be sensitive to this possibility.

## Conclusion

A more holistic approach to advising the traveller with cancer reflects the increased survivorship and expectations for a normal quality of life in this community. Although the infectious disease risks associated with immunosuppressive therapy are central to their preparation, we advocate a broader consideration of non-communicable disease risks and a discussion of the health benefits of international travel. Future qualitative research should elucidate the lived travel health experiences of this vulnerable group of travellers.

## Funding

This research did not receive any specific grant from funding agencies in the public, commercial, or not-for-profit sectors.

## Conflict of interests

The authors have no conflicts of interest to declare.
